# Chloroform and Cod Liver Oil in Diptheritus and Scarlatina

**Published:** 1860-03

**Authors:** 


*Chloroform and Cod Liver Oil in Diphtheritis and Scarlatina.*

We clip the following as communicated to the *Boston Med. and Surg. Journal*, by E. S. Cooper, M. D., Prof. of Anatomy and Surgery in the Medical Department of the University of the Pacific.

"The original mortality attending diphtherite, both in this country and Europe, appears to have been but little diminished by any course of treatment in general use. In some regions of country two in three cases terminate fatally; in others four in five; and in some localities of the United States probably there is even still greater fatality.

The first two cases I attended, both in one family, died. They were among the first patients I treated on my arrival in this city, over four years ago. Such was my ill success in attempting to cure these cases for some time afterwards, that I thought seriously of abandoning the treatment of the disease altogether. I had noticed, from the commencement of my practice in this disease, that the disposition to slough was always greater after I had used the probang. I attributed this partly to the effects of pressure, or bruising, incidental to the use of the instrument in young children, who are disposed to struggle. I have therefore abandoned all applications to the throat, and adopted the following method, which has thus far been successful beyond what my most sanguine hope could have anticipated. Of thirty-one patients, I have lost but one, and in that case the patient had been sick for several days, and died about eight hours after I first saw him.

My treatment is as follows: ℞.—Chloroform, ʒ iij.; ol. jec. aselli, ʒ xii.; spt. terebinth., ʒ ij. M. Signa. Apply freely all over the neck, breast, and abdomen, upon flannels covered with oil silk. This I keep on constantly during the continuance of the disease, and for eight or ten days after the patient has sufficiently recovered to walk about. The object of continuance is to prevent relapses, which are very frequent and fatal, without some preventive is used. And this is what is wanted in these cases. Internally, I direct the following to be administered: ℞—Ext. glycyrrh., ʒ iij.; acacia gum., ʒ i.; antimon. tart., gr. i.; sac. alb., ʒ ij.; aqua, ℥ xviij. M. Signa. Give a wine-glassful every two hours to a young child, say two years old, and increase in proportion to age. I have had as much, if not more, satisfaction in the results of the treatment of diphtherite on the foregoing plan, than in anything occurring in my professional life besides. I therefore recommend it with confidence to the medical profession. I have tried it with nearly the same success in scarlatina. During the course of treatment I do not give patients a particle of anything else; not a drop of water, nor the least nourishment, save what is in the medicine.

The compound keeps the bowels merely soluble, alleviates the cough, dryness in the throat, and difficulty of swallowing. I have, in some instances, added a grain or two of tartrate of antimony to the compound, and occasionally omit it altogether. I am convinced that certain states of the atmosphere increase the malignancy of diphtherite, and that chloroform and cod-liver oil annul its effects almost entirely. The oil protects the skin, and the chloroform acts probably upon the air-passages, while the turpentine acts as a counter-irritant. I have been surprised, and highly gratified, by noticing the rapidity with which the stoppage of the nostrils and difficulty of breathing are often overcome through this agency, even in advanced stages of the disease."

